# AI in Clinical Decision Support Systems: Promising Applications and Strategies for Managing Data Challenges

**DOI:** 10.2196/71532

**Published:** 2026-05-04

**Authors:** Jennifer E Daly, Dursun Delen, Zheng Han, River Smith, Jacqueline Honerlaw, Kelly Cho, Bridget Bennett, Jennifer Sippel

**Affiliations:** 1 Office of the Chief of Staff, Eastern Oklahoma VA Health Care System, 1101 Honor Heights Drive, Muskogee, 74401, United States, 1 757-784-5104, 1 757-781-1077; 2 Department of Management Science and Information Systems, Spears School of Business, Center for Health Systems Innovation, Oklahoma State University, Stillwater, OK, United States; 3 Faculty of Engineering and Natural Sciences, Department of Industrial Engineering, Istinye University, Sariyer, Istanbul, Turkey; 4 Department of Biomedical Engineering, University of Central Oklahoma, Edmond, OK, United States; 5 Office of the Associate Chief of Staff for Research, Eastern Oklahoma VA Health Care System, Muskogee, OK, United States; 6 Division of Population Health and Data Science, Massachusetts Veterans Epidemiology Research and Information Collaborative, VA Boston Healthcare System, Boston, MA, United States; 7 Division of Aging, Mass General Brigham, Harvard University, Cambridge, Boston, MA, United States; 8 Spinal Cord Injuries and Disorders National Program Office, United States Department of Veterans Affairs, Washington, DC, United States

**Keywords:** artificial intelligence, AI, delivery of health care, decision-making, machine learning, precision medicine, big data, population health, decision support systems, clinical medical informatics

## Abstract

The translation of big data analytics and artificial intelligence (AI) into clinical decision support systems (CDSSs) has advanced from proof of concept to real-world clinical practice. AI-informed CDSSs show measurable improvements in diagnostic accuracy, risk stratification, resource use, and patient outcomes compared to traditional models, offering the potential to assist clinicians in managing symptom complexity and uncertainty in health care delivery. Despite this potential, access to large amounts of high-quality and granular data remains one of the most significant bottlenecks to AI-enabled CDSSs. We argue that as health care systems increasingly adopt data-driven decision support, addressing the challenges of data accessibility and protection is essential to realizing the full potential of AI in clinical medicine. We use selected case examples of AI-informed CDSSs in oncology, organ transplantation, diabetic retinopathy, epilepsy, spinal cord injury, rare disease diagnosis, and emergency medicine to illustrate opportunities and challenges related to AI’s potential to improve patient outcomes. We discuss public and semipublic, medical institutional and commercial, and government and national data sources that are currently available for the development of CDSSs and highlight the practical and ethical constraints associated with these data. We consider alternative data resources and ways in which health care systems can strengthen data ecosystems to increase AI-driven CDSS efficacy and implementation to improve patient outcomes.

## Introduction

Artificial intelligence (AI) and big data analytics have emerged as powerful tools for medical decision support, offering the potential to augment clinicians’ diagnostic accuracy, improve prognostic estimation, and support more timely and personalized clinical decisions. AI-based clinical decision support systems (CDSSs) leverage large volumes of clinical, imaging, patient-generated, and administrative data to assist clinicians in managing symptom complexity and uncertainty in their delivery of health care.

Early CDSSs emphasized rule-based models intended to reduce errors and improve adherence to evidence-based care [1]. As electronic health records (EHRs) became more widely available, machine learning (ML) models were found to match or exceed clinician performance in domains such as medical imaging interpretation, early detection of clinical deterioration, and prediction of disease progression [2,3]. Natural language processing techniques further extended decision support by extracting clinically meaningful information from unstructured EHR data, enabling more comprehensive risk stratification and care planning [4]. Systematic reviews suggest that these tools may reduce cognitive burden and variability in decision-making while improving efficiency [5,6].

Recent research documents a maturation of AI-enabled CDSSs with a focus on real-world implementation. Studies highlight advances in explainable AI, clinician-AI interactions, and evaluation frameworks that move beyond algorithmic accuracy to assess clinical usefulness and workflow integration [5,6]. These studies identify key determinants of clinician use and trust, establishing factors such as transparency, usability, workflow integration, and training as central to adoption [7,8]. A meta-analysis grounded in the unified theory of acceptance and use of technology revealed that performance expectancy, effort expectancy, social influence, and facilitating conditions significantly influence health care practitioners’ intentions to use AI-enabled CDSSs [8]. Concurrently, research emphasizes the importance of explainable AI methodologies to support interpretability and user confidence, as well as human-centered evaluation frameworks that align technical designs with real-world clinical needs [6,9,10]. Additional work explores persistent problems and barriers associated with implementation, including technical fit, perceived risk, and task alignment, suggesting that successful CDSS integration hinges not only on algorithmic performance but also on organizational, human, and contextual factors [7,11-13]. These studies collectively underscore that while AI-driven CDSSs have strong potential to enhance clinical decision-making, their effective deployment requires attention to human factors, trust, usability, and structured implementation strategies.

The literature also identifies substantial challenges that limit the widespread and equitable adoption of AI in medical decision support. In addition to concerns about interpretability, bias, and workflow integration, access to high-quality data remains a critical barrier. Many health care systems face fragmented data infrastructures, restrictive data governance policies, and privacy regulations that limit data sharing across institutions [14]. Research has shown that AI models trained on narrow or nonrepresentative datasets may perform poorly when deployed in diverse clinical settings, exacerbating disparities in care [15]. Furthermore, smaller or resource-limited organizations often lack the technical capacity or data volume required to develop or adopt robust AI systems.

**Figure 1.**
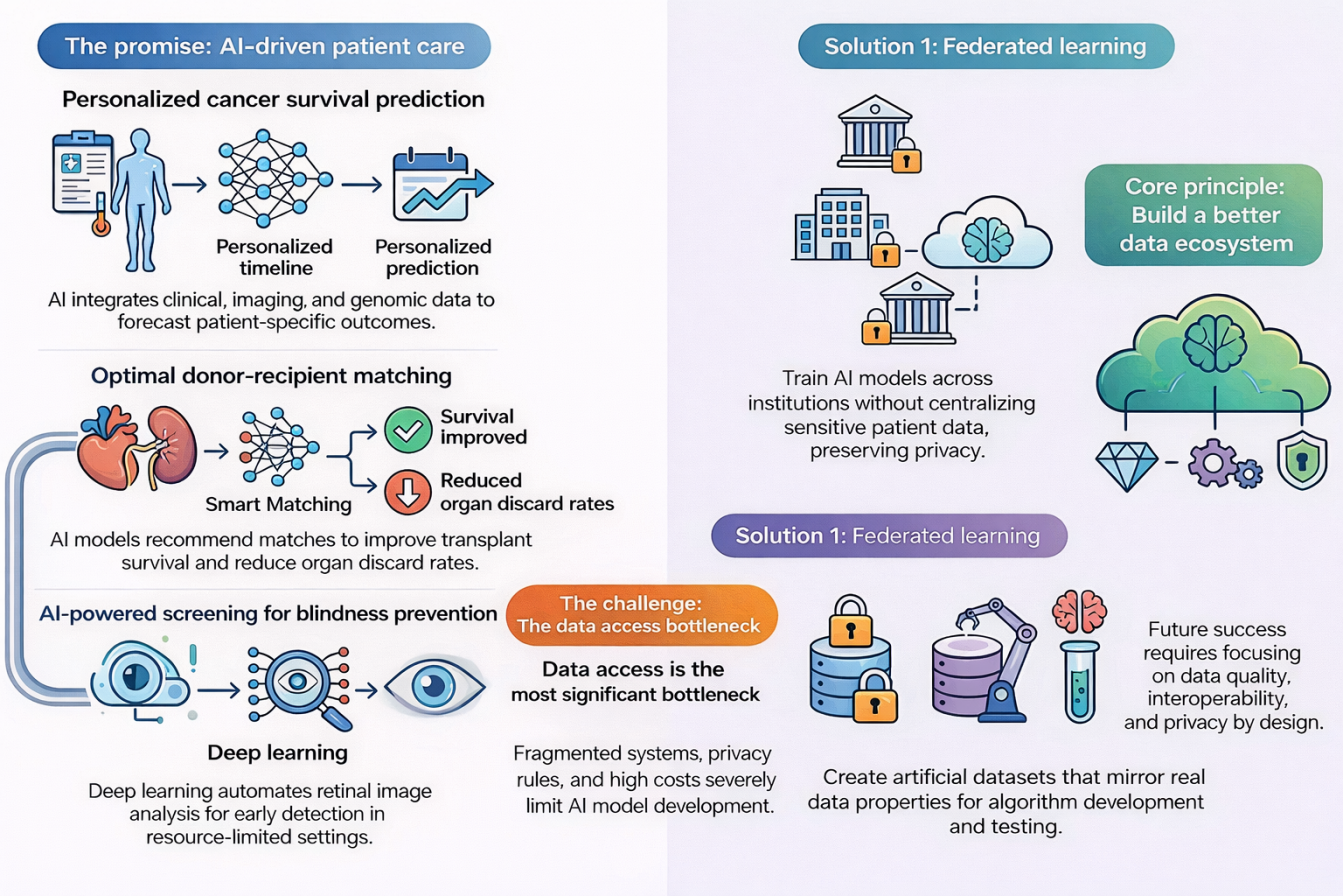
Visual overview of artificial intelligence (AI)–informed clinical decision support for selected case examples and potential solutions to overcome data access challenges.

This paper aims to clarify both the promise of AI-driven medical decision support and the structural data access challenges that must be addressed to realize its full clinical value. We argue that as health care systems increasingly adopt data-driven decision support, addressing the challenges of data accessibility and protection is essential to realizing the full potential of AI in clinical medicine. We review case studies and discuss synthetic data and federated networks as potential solutions for working with sensitive data. We offer a set of core principles to guide the development of hybrid data ecosystems for AI-enabled CDSSs that can make high-quality data easy to discover, use, and trust. Figure 1 provides a visual overview of the promise and challenges of AI-enabled CDSSs and potential solutions, which are discussed in greater detail in this paper.

## Data Access and Challenges of AI-Enabled CDSSs

### Selected Applications

The translation of big data analytics and AI in CDSSs has moved from proof of concept to real-world clinical impact. Data-driven CDSSs demonstrate measurable improvements in diagnostic accuracy, risk stratification, resource utilization, and patient outcomes across multiple areas, including oncology, organ transplantation, chronic disease management, emergency medicine, rare disease diagnosis, and spinal cord injury. These case studies illustrate the opportunities and challenges in deploying AI-enabled CDSSs in clinical medicine.

### Oncology: Personalized Cancer Characterization and Survival Prediction

Cancer care has been among the earliest and most fertile domains for AI-driven CDSSs due to the availability of high-dimensional data, including clinical variables, imaging, genomic profiles, and longitudinal outcomes. Early work demonstrated that ML models could outperform traditional statistical approaches in classifying cancer subtypes and predicting survival, laying the foundation for modern precision oncology [16].

Recent studies have advanced this paradigm by integrating heterogeneous data sources to enable patient-specific survival prediction and explainable risk stratification [17]. Deep learning and ensemble models have been used to uncover latent patterns across tumor biology, treatment response, and demographic factors, enabling individualized prognostic assessments rather than population-level averages [18]. Importantly, contemporary research emphasizes model interpretability, using explainable AI techniques to identify which clinical or molecular factors drive predictions, thereby increasing clinician trust and facilitating shared decision-making [19-21].

AI-driven CDSSs support oncologists’ treatment selection, risk-adjusted follow-up scheduling, and identification of high-risk patients who may benefit from aggressive or experimental therapies. However, challenges remain, including data imbalance across cancer subtypes, model generalizability across institutions, and ethical concerns surrounding algorithmic bias when predictions influence life-critical decisions.

### Organ Transplantation: AI-Enabled Optimal Donor-Recipient Matching

Organ transplantation presents a high-stakes optimization problem where mismatches can lead to graft failure, patient morbidity, and wasted organs. Traditional allocation systems rely on rule-based scoring methods that may not fully capture complex, nonlinear interactions among the donor, the recipient, and contextual factors [22].

Recent AI-driven CDSS approaches apply ML, optimization algorithms, and predictive analytics to maximize graft utilization while improving posttransplant survival [20]. These systems analyze large registries of donor and recipient data, learning patterns associated with graft longevity, rejection risk, and long-term outcomes. By simulating multiple allocation scenarios, AI models can recommend matches that balance equity, urgency, and expected benefit more effectively than static scoring systems [21].

Notably, these approaches highlight the role of big data in addressing systemic inefficiencies—reducing organ discard rates and improving fairness in allocation. Nevertheless, their adoption faces regulatory, ethical, and transparency challenges, particularly when algorithmic recommendations conflict with established allocation policies or clinician judgment.

### Diabetic Retinopathy: AI-Based Screening in Resource-Limited Settings

Diabetic retinopathy (DR) is a leading cause of preventable blindness, yet early detection remains limited in rural and underserved regions due to shortages of ophthalmologists [23]. AI-enabled big data analytics has emerged as a transformative solution, leveraging deep learning models trained on large retinal image datasets to automatically detect DR with accuracy that is comparable to that of expert clinicians [24]. These CDSS applications enable scalable, low-cost screening at the point of care, allowing for early referral and intervention before irreversible vision loss occurs [25]. Their integration into primary care and mobile screening units demonstrates how big data and AI can bridge health care access gaps and improve population-level outcomes.

However, despite the promise of DR CDSSs, challenges persist regarding dataset representativeness, robustness across imaging devices, and clinical workflow integration. Additionally, regulatory approval and medico-legal accountability remain critical considerations when automated systems are used for diagnostic decision-making.

### Traumatic Brain Injury and Posttraumatic Epilepsy

Traumatic brain injury (TBI) is a major global health concern, affecting millions annually and often leading to severe neurological sequelae, including posttraumatic epilepsy (PTE) [26]. Predicting the risk of PTE among patients with TBI is crucial for effective prevention. ML models that consolidate longitudinal patient encounter data, disease history, and laboratory records have emerged as a promising alternative to radiological or pathological data for risk assessment [27-29]. However, traditional ML models face limitations, including data sparsity due to their reliance on Euclidean data structure [30], requiring imputation that can introduce bias [16,17], and challenges in representing temporal data despite the rich sequence-based information embedded in patient encounters [31,32].

As a solution, Ramamurthy et al [33] leveraged a heterogeneous graph attention network (HeteroGAT) on large-scale data from the Cerner Real-World Data. By encoding hospital visits as patient-to-diagnosis edges within the graph structure, this HeteroGAT model eliminates the need for imputation, efficiently compresses data, and retains temporal information. The trained model was shown to outperform traditional models in predicting PTE risk among patients with TBI.

### Spinal Cord Injury: Improving Rehabilitation Planning and Prevention

A growing body of evidence shows that AI and ML models improve the prediction of clinically meaningful outcomes after spinal cord injury or disorder (SCI) [34-37]. These models forecast Spinal Cord Independence Measure scores [34], gait recovery [36], and changes in American Spinal Injury Association Impairment Scale grade [37] more accurately than traditional methods, supporting improved risk stratification and individualized rehabilitation planning. Multimodal approaches integrating clinical notes, imaging, electrophysiology, biomarkers, and social determinant data have enabled explainable, clinician-facing tools that enhance both predictive performance and interpretability. Advances in neuromodulation and device-assisted rehabilitation further suggest complementary pathways in which ML-guided patient selection and dosing may increase real-world benefit [38-48]. Additional applications include algorithms to predict postoperative health-related outcomes and guide surgical decisions [38,39], AI-trained robotics to improve upper extremity function through noninvasive electrical stimulation in tetraplegia [41], and molecular discovery efforts targeting neural regeneration by modulating inflammatory processes at the site of SCI [42].

Despite benefits and advances, most SCI studies to date are retrospective, single center, or registry based, restricting external generalizability and increasing the risk of overfitting. Public SCI datasets are relatively small and heterogeneous, and although there is a greater amount of Veterans Health Administration (VHA) data, they can be fragmented across clinical systems, complicating efforts to build robust, interoperable models. Moreover, few studies extend beyond prediction to demonstrate whether ML-guided care improves long-term outcomes such as functional recovery, quality of life, or reduced health care use.

### Rare Disease Characterization: Accelerating Diagnosis and Treatment Pathways

Rare diseases such as systemic lupus erythematosus and Crohn disease often present with heterogeneous symptoms that evolve over time, which can lead to delayed or missed diagnoses. AI-driven CDSSs have shown promise in characterizing disease subtypes, identifying early diagnostic signatures, and predicting disease progression using longitudinal EHR data [49].

By mining structured and unstructured clinical data, these systems uncover subtle patterns that may elude traditional diagnostic approaches. Early and accurate diagnosis enables timely treatment initiation, improved symptom control, and reduced long-term complications—outcomes particularly critical in diseases in which diagnostic delays can span years [50].

The primary challenge with rare diseases lies in limited sample sizes and fragmented data, which complicate model training and validation. Federated learning and multi-institutional data sharing, which are discussed in later sections, are emerging as potential solutions to overcome these barriers while preserving patient privacy.

### Emergency Medicine: Reducing Emergency Department Bounce Backs Using Unstructured Data

Emergency department (ED) bounce backs—unplanned return visits shortly after discharge—are costly, burdensome, and often indicative of missed diagnoses or inadequate discharge planning. Recent CDSS research leverages natural language processing and ML to analyze unstructured clinician notes, capturing clinical nuance missing from coded data [51].

These systems identify patients at high risk of bounce back by modeling symptom descriptions, clinician assessments, and contextual factors documented in free text. By flagging high-risk cases in real time, CDSSs support more informed discharge decisions, targeted follow-up, and resource allocation, ultimately improving patient safety and reducing unnecessary ED use [52].

Key challenges with ED models include variability in clinical documentation, model explainability, and real-time integration into fast-paced ED workflows. Nonetheless, this case exemplifies the untapped value of unstructured EHR data in advancing AI-driven clinical decision support.

### Summary of AI-Enabled CDSS Applications

Collectively, these case studies demonstrate that big data and AI-enabled CDSSs can meaningfully improve diagnosis, prevention, risk stratification, and outcomes across diverse clinical domains. However, their success depends not only on algorithmic sophistication but also on data quality, interpretability, ethical governance, and seamless clinical integration. As health care systems increasingly adopt data-driven decision support, addressing these challenges is essential to realizing the full potential of AI in clinical medicine.

## Big Data Resources for AI-Enabled CDSSs

### Overview

Despite the demonstrated value of AI and advanced analytics for improving health care decision-making and patient outcomes, access to large amounts of high-quality and granular clinical data remains a major bottleneck. Health care data are inherently sensitive, fragmented across institutions, governed by complex regulatory frameworks, and costly to curate and integrate. Although a small number of public and semipublic datasets have enabled important advances in AI-enabled CDSSs, most clinically rich datasets remain difficult to access for scientific and translational research.

### Public and Semipublic Health Care Datasets: Opportunities and Limitations

Several large-scale datasets have played a foundational role in advancing AI-driven CDSSs by providing researchers with access to real-world patient data at scale.

The Surveillance, Epidemiology, and End Results Program provides longitudinal cancer incidence, treatment, and survival data across 22 US geographic regions, representing approximately 48% of cancer cases nationwide and supporting population-level oncology research [53].

The United Network for Organ Sharing maintains a comprehensive transplant registry containing data on all US organ donation and transplant events since 1987, enabling modeling of donor-recipient matching and graft survival.

The Centers for Medicare and Medicaid Services (CMS) databases offer large administrative claims datasets for studying health care costs, quality, and outcomes. CMS uses a tiered access model that includes publicly available synthetic public use files for exploratory analyses [54] and fee-based access to patient-level data through the Research Data Assistance Center, requiring data use agreements, institutional review board (IRB) approval, and additional safeguards for limited or identifiable datasets [55,56]. This multitiered model demonstrates a pragmatic approach to data democratization that lowers barriers for exploratory research while maintaining rigorous governance for sensitive analyses.

### Medical Institution–Based Data Warehouses and Commercial Real-World Data Platforms

To address the limitations of public datasets, researchers have turned to medical institution–managed and commercial real-world data (RWD) repositories, which aggregate richer EHR data across multiple health care systems. These platforms offer greater clinical granularity—including clinical notes, laboratory results, medications, imaging metadata, and care pathway details—enabling more nuanced AI-driven CDSS development and validation. However, their data sharing models, access mechanisms, and computational infrastructures differ markedly from those of government-sponsored repositories, presenting both opportunities and challenges for researchers.

Oracle Real-World Data (ORWD), formerly Cerner Real-World Data, is one of the largest commercial aggregators of real-world evidence, curating deidentified EHR data from over 100 million patients across diverse health care settings in the United States and internationally [57]. The platform integrates claims data, laboratory results, clinical documentation, and medication records from hospital systems, ambulatory clinics, and specialty care centers. ORWD is distinguished by its data sharing and analytics infrastructure designed for pharmaceutical research, regulatory submissions, and comparative effectiveness studies. Access is governed by subscription-based licensing and data use agreements that define permitted research questions, data elements, cohorts, and publication rights; access is often limited to organizations that contribute data to Oracle Health. ORWD provides a secure, cloud-based analytical environment hosted on Amazon Web Services where approved researchers access harmonized datasets without direct download to maintain privacy. The platform supports SQL-based tools (eg, Spark) and R and Python integrations, enabling large-scale model development and validation using distributed computing resources.

The Epic Systems Cosmos platform has emerged as a powerful force in RWD aggregation, leveraging Epic’s market position as the leading EHR vendor in the United States [58]. As of 2024, Cosmos contains deidentified records from over 300 million patients across more than 1000 health care organizations. Similar to ORWD, Epic’s data sharing structure operates through a collaborative research network model in which participating health systems agree to contribute data in exchange for access. Notably, Cosmos has enabled large-scale studies on COVID-19 outcomes [59], opioid prescribing patterns [60], and other health issues. Cosmos represents another pragmatic example of how EHR vendors such as Oracle Health can leverage their market position to create valuable research infrastructure while balancing commercial interests with public health benefit.

Collectively, ORWD and Epic Cosmos illustrate a commercial data sharing paradigm that contrasts sharply with government-sponsored open access models. These platforms offer exceptional clinical granularity, scale, and near–real-time data availability, enabling research that would be infeasible with traditional registry or claims-based datasets. Their cloud-based analytical environments and integrated development tools lower technical barriers for approved researchers, facilitating rapid cohort identification and model development. However, subscription-based or partnership-driven access models introduce substantial financial and institutional barriers, limiting participation to well-resourced organizations and raising equity concerns. While these platforms provide access to structured data and, in some cases, unstructured clinical notes to support AI-driven CDSS development, access remains constrained by membership requirements, high costs, contractual restrictions, institutional review processes, and governance constraints. Data harmonization across sites also remains a significant challenge, limiting model portability and reproducibility.

### Government and National Health System Data Repositories

National and government-sponsored initiatives also offer uniquely rich datasets for model development and clinical research.

**Figure 2.**
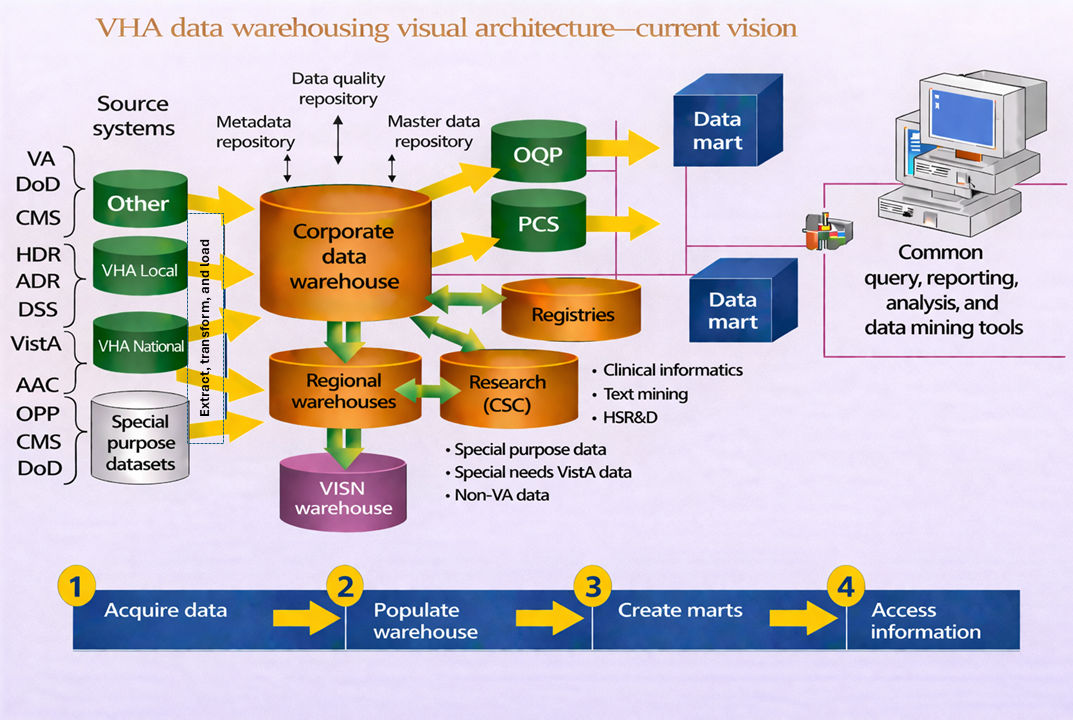
Veterans Health Administration (VHA) data warehousing architecture. AAC: Austin Automation Center; ADR: Administrative Data Repository; CMS: Centers for Medicare and Medicaid Services; CSC: Clinical Studies Center; DoD: Department of Defense; DSS: decision support system; HDR: Health Data Repository; HSR&D: Health Services Research and Development; OPP: Office of Planning and Programs; OQP: Office of Quality and Performance; PCS: Office of Patient Care Services; VA: US Department of Veterans Affairs; VISION: Veterans Integrated Service Network; VistA: Veterans Health Information Systems and Technology Architecture.

Veterans Health Affairs has a robust and integrated data infrastructure that supports clinical care, operational decision-making, and research across the VHA. Central to this infrastructure is the US Department of Veterans Affairs Informatics and Computing Infrastructure (VINCI), a resource of the Veterans Affairs (VA) Office of Research and Development that provides researchers with nationwide access to VA patient data [61]. VINCI partners with the Corporate Data Warehouse (CDW), which aggregates health care–relevant clinical and operational data from across the VHA into a relational database to facilitate enterprise-level reporting, performance measurement, management decision-making, and research. In addition to hosting all CDW data, VINCI provides unique longitudinal views of patient care and supports advanced analytics through the Observational Medical Outcomes Partnership Common Data Model. The Observational Medical Outcomes Partnership Common Data Model is technology neutral and optimized for large-scale computational analysis, enabling evaluation of associations between interventions and outcomes across datasets containing millions of patients and billions of clinical observations. To support data reuse and reproducibility, standardized phenotypes with documented curation methods and decision rules are maintained in the VA’s Centralized Interactive Phenomics Resource knowledge base [62,63]. Figure 2 illustrates the current architecture of VHA data.

Complementing the VINCI and CDW are specialized VHA registries that enable in-depth study of rare, complex, and veteran-specific conditions. These registries include detailed longitudinal clinical assessments and EHR data for veterans receiving specific treatments or follow-up care, such as the VA Central Cancer Registry, the Health Outcomes Military Exposures registry—which encompasses multiple exposure-related surveillance programs—and the VA Spinal Cord Injuries and Disorders Registry [64-66]. Patients are tracked over time and remain in registries even after death, supporting epidemiologic research and monitoring of disease incidence, treatment patterns, care efficiency, outcomes, and costs. Access to VA data is restricted to VA employees and non-VA researchers who obtain without-compensation employee status and complete a formal approval process, including submission of a data management plan, IRB approval, and research protocols through VINCI.

The strength of VHA data lies in the size of patient cohorts; their depth and granularity; and their longitudinal nature, which allows for tracking changes over time, establishing causality and temporal dynamics, controlling for individual differences, increasing statistical power, understanding developmental processes, and reducing bias. However, although VA data are notably rich and more complete than many civilian datasets, they are limited by data quality, such as inaccuracies, missing data, and inconsistencies, as well as the complexity of integrating data from various sources with differing formats and standards (eg, the US Department of Defense, CMS, individual VHA facilities, and overall national VHA data). Additionally, ensuring data privacy and security, managing the vast volume of data, addressing potential biases and representativeness issues, and requiring advanced technical expertise and computational power can present significant hurdles to effective big data analytics and AI implementation.

The UK Biobank offers one of the most comprehensive population-scale datasets worldwide, integrating EHRs, imaging, genomics, lifestyle data, and long-term follow-up [67]. Launched in 2006, it enrolled over 500,000 participants aged 40 to 69 years across the United Kingdom, collecting extensive health records, genomic data, multimodal imaging (including brain, cardiac, and abdominal magnetic resonance imaging), lifestyle questionnaires, physical measurements, and longitudinal outcomes via linkage to national registries. Beyond its scale, the UK Biobank is distinguished by its transparent open access model designed to maximize global research utility while maintaining participant trust. Approved researchers worldwide can apply through a streamlined online process, with applications reviewed solely based on scientific merit and feasibility rather than institutional affiliation or commercial interests [68]. Researchers execute a material transfer agreement, commit to peer-reviewed publication, return derived data and algorithms, and submit annual progress reports. Data access is subject only to nominal cost-recovery fees, and research outputs must be publicly available, fostering transparency and reproducibility. The application-to-access timeline typically spans weeks, accelerating the research life cycle. As of 2024, the UK Biobank has supported over 9000 peer-reviewed publications across diverse disease domains, including genome-wide association studies, cardiovascular risk prediction models, and AI-based retinal image analyses for systemic disease detection [68-71]. This model demonstrates that large-scale data sharing, coupled with robust yet nonburdensome governance and privacy protections, can accelerate discovery without compromising ethical stewardship [72].

The National Institutes of Health All of Us research program exemplifies a shift toward inclusive, participant-centered precision medicine in the United States, emphasizing innovative data access infrastructure [73]. Launched in 2018 to enroll 1 million diverse participants, All of Us addresses the underrepresentation of diverse populations in large-scale health studies. The program collects EHRs, genomic data, biospecimens, wearable sensor data, and self-reported health information, creating a rich, longitudinal dataset reflecting US diversity. A key feature is its cloud-based, open science data sharing model through the Researcher Workbench—a secure, scalable platform democratizing data and computational tool access. The Workbench operates on a tiered access system: a public tier with deidentified aggregate data; a registered tier with deidentified, aggregated data for researchers upon completing ethics training; and a controlled tier with individual-level genomic and detailed data for researchers with additional credentials and IRB approval. The platform reduces privacy risks by providing analysis tools without requiring data download, enabling research using preconfigured Jupyter notebooks and R and Python environments [73].

All of Us prioritizes participant engagement and data return, providing participants with access to their genetic results and health summaries, thereby fostering transparency and trust [74]. This bidirectional data flow respects participant autonomy while maximizing public benefit. Recent studies using All of Us data have advanced genetic diversity understanding in pharmacogenomics, identified novel disease associations, and validated AI-driven clinical models across diverse demographics [74,75]. By emphasizing diversity, participant empowerment, accessible infrastructure, and open science, the All of Us research program advances precision medicine and models an ethically grounded, technologically sophisticated approach to big data governance. Initiatives such as this one, alongside the UK Biobank and CMS’s tiered model, demonstrate that rigorous, transparent data sharing and collaboration can transform global health care delivery and outcomes. These national and government-sponsored data resources demonstrate the power of centralized, well-governed data infrastructures, but access is often limited by citizenship, institutional affiliation, or strict use agreements, which reduces global accessibility and, ultimately, affects their potential for use in the development and continuous improvement of AI-enabled CDSSs.

## Emerging Alternatives and Privacy-Preserving Solutions for Practical and Ethical Challenges

### Overview

Using EHR data and AI to predict health outcomes presents practical challenges, particularly given patients’ vulnerabilities and the need for equitable access to high-quality care. Medical data are often fragmented across systems, incomplete, or inconsistently documented, limiting the accuracy and generalizability of predictive models. Patients may also have unique or context-specific health profiles, and models trained on historical data may fail to reflect new treatments, evolving standards of care, or shifting disease patterns. Additional limitations include “truth in data” issues—such as the use of diagnostic codes to rule out rather than document conditions—and collider bias, which can distort model outputs [76,77]. Furthermore, the opaque or “black box” nature of many ML models can undermine clinician trust and impede adoption in high-stakes clinical settings where understanding the rationale behind predictions is essential.

Ethical concerns further complicate the use of AI in clinical decision-making, particularly related to bias, fairness, and privacy. Training datasets that underrepresent certain populations, such as women or racial and ethnic minority groups, risk perpetuating inequities in care. Privacy risks are heightened because medical data are highly sensitive and vulnerable to breaches, and overreliance on algorithmic predictions may unintentionally diminish clinician judgment and patient-centered care. Addressing these challenges requires deliberate safeguards, transparency, and accountability, as well as continued involvement of clinicians and patients in system design and use. Balancing the benefits of AI with the protection of patient rights, dignity, and equity is essential to the responsible integration of AI and ML into precision medicine. Given these ethical and legal constraints surrounding patient data, several alternative approaches are gaining traction.

### Synthetic Data Generation

Advances in generative AI now enable the creation of high-fidelity synthetic health care datasets that preserve statistical properties and clinical relationships while minimizing reidentification risk. When carefully validated, synthetic data can support algorithm development, benchmarking, and educational use. However, challenges remain in ensuring clinical realism, avoiding hidden biases, and validating downstream model performance.

The main advantage of synthetic data is that they provide a dynamic, data-driven simulation of real-world systems, which can enable prediction, optimization, and decision-making without violating privacy or disrupting patient care. However, because synthesized data rely on collected and modeled data to mirror a real system, they are only as reliable as the quality, level of detail, and timeliness of the source data. Incomplete, biased, or outdated inputs can lead to misleading outputs, reinforcing poor decisions rather than improving them. There is also a risk of overfitting, where the model learns patterns that fit past data too closely, making it less able to handle new or unexpected situations. Additionally, maintaining a synthetic dataset is resource intensive, requiring constant synchronization, governance, and security oversight—making them vulnerable to drift, privacy risks, and escalating costs. Synthesized patient data also raise privacy concerns, as building detailed digital counterparts often requires access to sensitive or personal data that can be exposed or misused if not properly protected. Finally, synthetic data struggle with generalization: insights derived from one context may not transfer well to different environments, contributing to misleading conclusions when applied outside the conditions under which the synthesized dataset was trained or calibrated. All these concerns must be considered before such data can inform policy or treatment decisions.

### Federated Data Networks

Federated learning enables collaborative AI development across health care institutions without centralizing sensitive patient data. Instead of aggregating data in one place, a federated learning algorithm sends the model to each site, where institutions train it locally and share only parameter updates—not raw records that need to be constrained within institutional firewalls. A central server aggregates these updates (eg, via federated averaging) to iteratively refine a global model. This process continues until convergence, producing a model that learns from multi-institutional data while maintaining privacy and data sovereignty. This approach is particularly promising for rare diseases, multicenter studies, and regulatory-compliant AI development, although it introduces technical complexity and coordination overhead.

However, federated learning networks are limited by diverse organizational and technical landscapes, infrastructure variances, and differences in secure processing environments, as well as the extent of data harmonization, fragmentation, heterogeneity across data sites, and generalizability. Addressing these challenges would put tremendous strain on the existing IT infrastructure. Nonetheless, federated data provide a way for researchers to access extensive patient data across various locations to design algorithms with high accuracy, broad applicability, and minimal resource utilization.

## Proposed Framework for Synthesis: Balancing Access, Privacy, and Innovation

**Figure 3.**
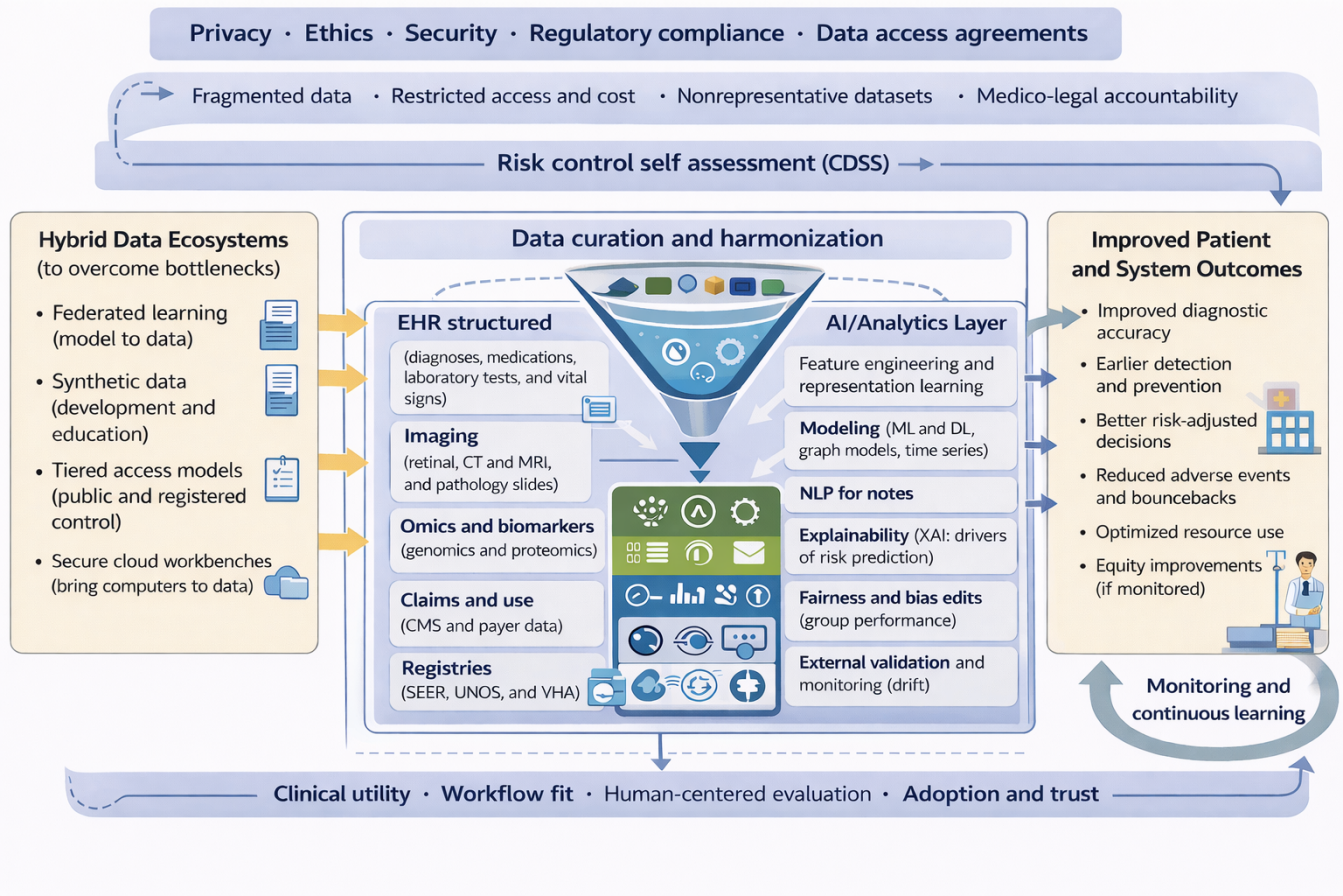
Proposed framework for overcoming data bottlenecks to improve patient and system outcomes. AI: artificial intelligence; CDSS: clinical decision support system; CMS: Centers for Medicare and Medicaid Services; CT: computed tomography; DL: deep learning; EHR: electronic health record; ML: machine learning; MRI: magnetic resonance imaging; NLP: natural language processing; SEER: Surveillance, Epidemiology, and End Results; UNOS: United Network for Organ Sharing; VHA: Veterans Health Administration; XAI: explainable AI.

AI holds immense potential to transform health care by providing data-driven decision support, optimizing workflow, and improving patient outcomes. However, the tension between data accessibility and data protection defines the current landscape and constrains the development of AI-driven clinical health care models. While public datasets and national repositories provide essential foundations, they are insufficient on their own to support the next generation of data-driven CDSSs. Scalable progress requires a hybrid ecosystem that combines curated RWD, synthetic data augmentation, and federated architectures that are supported by clear governance frameworks and incentives for responsible data sharing. Figure 3 illustrates a detailed outline for a framework to overcome data bottlenecks and improve patient and system outcomes.

Building hybrid data ecosystems for AI-enabled CDSSs requires a reliable, secure, and well-governed foundation that makes high-quality data easy to discover, use, and trust. Core principles include the following:

- Quality over quantity—establish strong data quality standards, validation, and continuous monitoring. For AI systems, this includes validating ground truth labels and detecting and correcting systematic errors in data collection.
- Interoperability and reusability—use open table formats and common vocabularies so data can move across tools and teams. Adopt standard instruments and frameworks for clinical data with consistent preprocessing and design schemas.
- Privacy and ethics by design—include consent, deidentification, and access controls into pipelines rather than as afterthoughts or preuse requirements. Monitor performance across demographic groups to continuously assess bias and fairness.
- Observability and lineage—trace where data came from, how they were transformed, and how they impact models to increase transparency and explainability, making processes understandable to clinicians and affected individuals.
- Self-service with guardrails—empower users to safely discover, understand, and request data via catalogs and data products. Establish data stewards with clear roles and responsibilities, definitions, quality rules, and catalog curation. Use data protection impact assessments to audit compliance, security, and privacy.

Ultimately, overcoming data availability challenges is not merely a technical problem—it is an organizational, regulatory, and ethical one. Addressing these barriers will be essential to translating AI innovation into sustained improvements in patient outcomes.

## Ethical Considerations

This study did not involve human participants or identifiable data, so ethics approval was not required. Studies completed by other researchers and reviewed in this paper were conducted in accordance with the ethical standards of the institutional review boards of affiliated institutions. All data used in these studies were deidentified and compliant with the 1996 HIPAA (Health Insurance Portability and Accountability Act) regulations to ensure patient privacy and confidentiality. The ethical considerations related to secondary use of existing health care data for AI research were rigorously upheld in all studies conducted by the authors of this paper.

## Abbreviations

AI: artificial intelligence
CDSS: clinical decision support system
CDW: Corporate Data Warehouse
CMS: Centers for Medicare and Medicaid Services
DR: diabetic retinopathy
ED: emergency department
EHR: electronic health record
HIPAA: Health Insurance Portability and Accountability Act
IRB: institutional review board
ML: machine learning
ORWD: Oracle Real-World Data
PTE: posttraumatic epilepsy
RWD: real-world data
SCI: spinal cord injury or disorder
TBI: traumatic brain injury
VA: United States Department of Veterans Affairs
VHA: Veterans Health Administration
VINCI: United States Department of Veterans Affairs Informatics and Computing Infrastructure
